# Outcomes Used for the Evaluation of Mental Health Helplines: A Systematic Review

**DOI:** 10.1192/bjo.2024.488

**Published:** 2024-08-01

**Authors:** Sajid Mahmood, Damodar Chari, Mohamed Hamid, Rashid Khan, Pam Pannilavithana

**Affiliations:** 1 Leicestershire Partnership NHS Trust, Leicester, United Kingdom; 2 Leicester Medical School, Leicester, United Kingdom

## Abstract

**Aims:**

Helplines and crisis lines are a standard component of a public health approach which appear to be intuitively supportive and useful to a population in acute distress and prevent severely adverse outcomes i.e., suicide. These services exist in different formats throughout the world. They have the advantage of being widely accessible, approachable, and bypass the waiting times and bureaucracies of referral systems for accessing secondary mental health services. The authors set out to study the range of outcomes used to evaluate mental health helplines and crisis lines. The focus was not simply to explore whether mental health helplines were effective or not. Rather the authors wanted to investigate what outcomes were being considered as evidence.

The authors aimed to conduct a systematic review of evidence for mental health outcomes of service users of helplines and crisis lines.

The research question was, ‘What outcomes are evidenced in published literature for mental health helplines and/or crisis lines in terms of efficacy, effectiveness or efficiency?’

**Methods:**

This was a systematic review of literature using the PRISMA-2020 statement. Literature searches of Web of Science, Ovid (PsycINFO, Medline, EMBASE), PubMed and Scopus were conducted in December 2022. Relevant information from eligible studies was extracted by using a structured data extraction form. Mixed Methods Appraisal Tool (MMAT) was used to assess quality of the included studies. While the heterogeneity of studies prevented a meta-analysis, it provided a rich landscape for exploring the topic through a thematic analysis.

**Results:**

Eighteen studies finally met the inclusion and exclusion criteria. The projects studied used both trained professionals and volunteers trained to offer crisis intervention. Both qualitative and quantitative outcomes were evaluated across the studies. Outcomes were frequently subjective assessments of service users and/or the personnel delivering the intervention. Studies evaluated outcomes in various ways. Anonymity of the callers made long-term follow-up difficult in most cases, though it is understandable that anonymity might have contributed to the helpline being more accessible and less intimidating to the callers. MMAT scores showed the papers have a range of methodological soundness.

**Conclusion:**

There is lack of consensus and uniformity regarding what outcomes can evidence the efficacy, efficiency, and effectiveness of mental health helplines. Before more investment in helplines, there needs to be discussion, planning and understanding among policy makers and service developers in deciding what they want to achieve from a mental health helpline. This will help researchers focus on relevant outcomes to evaluate mental health helplines. Services need clarity regarding what difference they are trying to make when such helplines are set up.

